# Activity of Serpins in Context to Hydrophobic Interaction

**DOI:** 10.3390/biom15111615

**Published:** 2025-11-18

**Authors:** Irena Roterman, Katarzyna Stapor, Grzegorz Zemanek, Dawid Dulak, Leszek Konieczny

**Affiliations:** 1 Department of Bioinformatics and Telemedicine, Jagiellonian University—Medical College, Medyczna 7, 30-688 Krakow, Poland; 2 Faculty of Automatic, Electronics and Computer Science, Department of Applied Informatics, Silesian University of Technology, Akademicka 16, 44-100 Gliwice, Poland; 3 Chair of Medical Biochemistry, Jagiellonian University—Medical College, Kopernika 7, 31-034 Krakow, Poland; 4 ABB Business Sp z o. o., Zaganska 1, 04-713 Warszawa, Poland; dawid.dulak@gmail.com

**Keywords:** serpin, suicide inhibition, hydrophobicity

## Abstract

The activity of serpins uses a specific mechanism or process. This process comprises several steps and is related to significant structural changes that involve significant displacement of chain fragments and whole molecules of protease. An important role is played by a segment of the serpin chain called the Reactive Central Loop (RCL), which interacts with the protease by inhibiting its activity. For the covalent binding of the protease to serpin, the movement of the protease molecule is an effect of splicing the RCL segment into beta-sheet A of serpin. There are structural forms—native, latent, Michaelis complex (non-covalent enzyme-inhibitor complex prior to RCL cleavage), covalent serpin–protease complex, and cleaved—associated with serpin activity. In this work, all these structural forms are discussed using the fuzzy oil drop (FOD-M) model, where the assessment criterion of structuring is based on identifying the type of hydrophobicity distribution. The analysis reveals the specificity of the inhibition mechanism, including the specific action of the RCL. The structural changes involved in this process have been shown to preserve the distribution of hydrophobicity in the form preferred by the aqueous environment in which serpins are active. The disorder (according to FOD-M model) in two complexes (Michaelis and covalent) is hypothetically treated as code for degradation factors. The applied model assesses the function-related structures using the hydrophobicity distribution as the criterion in contrast to many publications based on energetic aspects of serpin activity. Structural changes appear appropriate for water environments—the environment of serpin activity.

## 1. Introduction

Maintained homeostasis is a critical condition for living organisms to continue to function [1,2]. It has therefore been a focus of many therapeutic searches [3]. A system with a preserved homeostasis is also an object of interest in mathematical analyses leading to the design of models that satisfy the stabilisation conditions [4,5,6]. Serpin-mediated inhibition has a special role in maintaining homeostasis [7]. The serpin family, which includes more than 1500 proteins, is tasked with ‘SERine Protease INhibition’ (hence the abbreviation ‘serpin’) [8]. Protease inhibition is particularly important when protease, a proteolytic enzyme, finds itself in the extracellular space, where its activity is no longer controlled. Here, the role of serpins, which inactivate protease, is proving critical [9,10,11,12,13,14]. The presence of serpins in non-cellular fluids—especially blood—is proportional to the presence of protease. Indeed, inhibition of protease activity is also associated with serpin inactivation, which is referred to as ‘suicide inhibition’ [15,16,17]. Serpin activity features a unique, complex mechanism of inhibition when reacting with protease [18,19,20,21,22,23,24]. Apart from its native form, the serpin molecule adopts very different structural forms because of the nature of the inhibition process; the possible forms can be latent, Michaelis complex (non-covalent enzyme-inhibitor complex prior to RCL cleavage), covalent serpin–protease complex, and cleaved. This is why the relation structure-function appears of special interest [25,26,27,28]. The influence of specific mutations on changes addressed to function-related positions in chain [29,30,31] as well as oriented on inhibition is discussed in [32]. The evolutional changes and their influence on function reveals the relationships to certain diseases [33,34,35]. The comparable inter-species analysis reveals the ubiquity of serpins activity [36,37,38,39,40,41]. The biological activity of serpins critical for organism functioning makes this group of proteins of high interest in medicine [42,43,44,45,46,47,48] and searching for therapy [49,50], especially in cancer [51] and COVID [52].

The CATH criteria [53] identify two domains in the structure of serpin: D1 and D2. A secondary structure is present in both domains and has the form of a beta-sheet. The beta-sheet of domain D1 is designated as ‘A’, and the beta-sheet of domain D2 is designated as ‘B’. The specifics of the protease activity inhibition lie in the generation of a non-covalent interaction with protease, which becomes a Michaelis complex [54]. Inhibition of the active centre of protease is deactivated by a non-covalent interaction with a serpin loop called the Reactive Central Loop (RCL). If the process of interaction between serpin and protease becomes protracted, the serpin chain is cleaved (with the cleavage site referred to as PI-PI’—(358–359)), leading to the formation of a covalent serpin–protease complex. This process inactivates the protease; however, serpin also loses its inhibitory capacity. As a result of cleavage, the C-terminal chain fragment of serpin remains a component of the D2 domain of the protein. In contrast, the released part of the RCL (upstream of the cleavage site) is displaced by incorporating itself into beta-sheet A together with the covalently bound protease. When a covalent serpin–protease complex is formed, serpin loses its activity. This is why this process is sometimes referred to as ‘suicide inhibition’ [23,54,55].

In this work, the structural forms of serpin will be analysed: native α1-antitrypsin (the active form), the latent form of serpin unstable intermediate (M*), the Michaelis non-covalent complex, the covalent serpin–protease complex, and cleaved serpin [54]. The status of the different structural forms will be discussed based on an analysis of the distribution of their hydrophobicity. Serpins operate in the environment of blood and therefore in an aqueous environment. The assessment of the state obtained in each form from the point of view of hydrophobicity distribution proves to be important and significant for the activity of the protein of interest. The stabilisation factor assumed to be a criterion is the assessment of hydrophobic interactions expressed as a form of hydrophobicity distribution in functionally diverse structural forms of serpin. Serpins are proteins that operate in an aqueous environment, so their status in relation to preferred structural forms for the aqueous environment is found to be important.

The analysis presented in this paper discusses the structural changes using the hydrophobicity distribution in protein/complex body. The presence of a hydrophobic core is treated as the stabilisation factor. The incorporation of the chain fragment (Reactive Central Loop) RCL to the beta-sheet is treated as specific form of domain swapping engaging chain fragments of the same protein molecule.

## 2. Materials and Methods

### 2.1. Data

A summary of the analysed structural forms of serpin—representing its diverse structural forms related to function—is provided in Table 1. Structures were analysed using same technology—X-ray with R-factor 0.193–0.231 and R-free 0.231–0.290.

**Figure 1:**
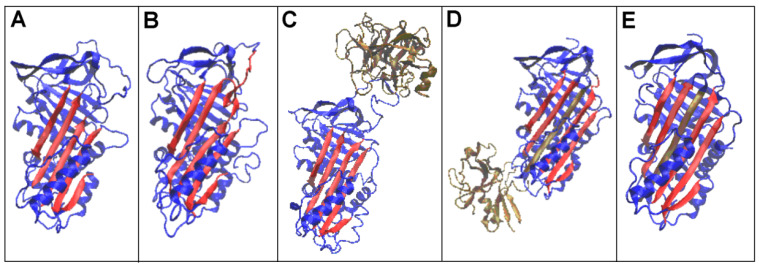
Three-dimensional presentation of the structures of interest with highlighted beta-sheet A—red. (**A**)—native (1QLP). (**B**)—latent (2ANT). (**C**)—Michaelis complex (serpin–trypsin) (1K9O) trypsin—ochre. (**D**)—acyl-enzyme complex (serpin–trypsin) (1EZX) trypsin—ochre, the chain fragment incorporated into beta-sheet A—ochre. (**E**)—cleaved (7API)—the chain fragment incorporated into beta-sheet A—ochre. The beta-sheet distinguished as red in (**A**–**C**) and red with one beta stands in ochre in (**D**,**E**) is called as beta-sheet A in this paper.

A summary of the forms analysed is also provided in Figure 1, where structural changes within beta-sheet A, consisting mainly of the adoption of an additional beta-strand into the beta-sheet composition, are depicted (Figure 1D,E—ochre). A change in the position of trypsin is also illustrated (Figure 1C,D—ochre)

**Figure 2:**
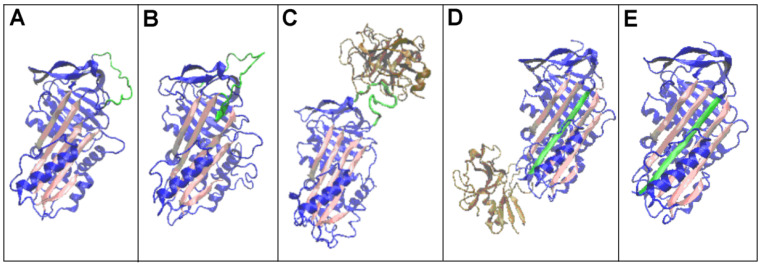
Three-dimensional presentation of the structures of interest with highlighted beta-sheet A—pink, and mobile reactive centre loop (RCL)—green. (**A**)—native (1QLP). (**B**)—latent (2ANT). (**C**)—Michaelis complex (serpin–trypsin) (1K9O) trypsin—ochre. (**D**)—acyl-enzyme complex (serpin–trypsin) (1EZX) trypsin—ochre. (**E**)—cleaved (7API)—the chain fragment incorporated into beta-sheet A—ochre.

Changes in the structural forms associated with the function concerning the reactive centre loop (RCL) are shown in Figure 2. (Figure 2—RCL—green).

The detailed analysis discussed here concerns changes in the status of the beta-sheet A (Figure 1—red fragments), a result of the RCL chain fragment presence in the structuring of the sheet (Figure 2—green fragment) and the role of the RCL in the biological activity of serpin. The data used for calculation to characterise the beta-sheets are given in Table 2.

### 2.2. Description of the Model Used

The accepted assessment criterion for the protein status was to evaluate the type of hydrophobicity distribution present in the protein structure. The distribution present in a protein—the result of inter-residual hydrophobic interactions—was assessed against two reference distributions: (1), representing the presence of an idealised centric hydrophobic nucleus and polar surface is referred to as ‘T’, and (2), representing an equal level of hydrophobicity that does not distinguish any position or any residue, is referred to as ‘R’. The proximity of the observed distribution, ‘O’, to one of the reference distributions allowed the presence/absence of a hydrophobic nucleus to be assessed.

A detailed description of the fuzzy oil drop model (FOD-M), which has been discussed many times, is provided in [58].

**Figure 3:**
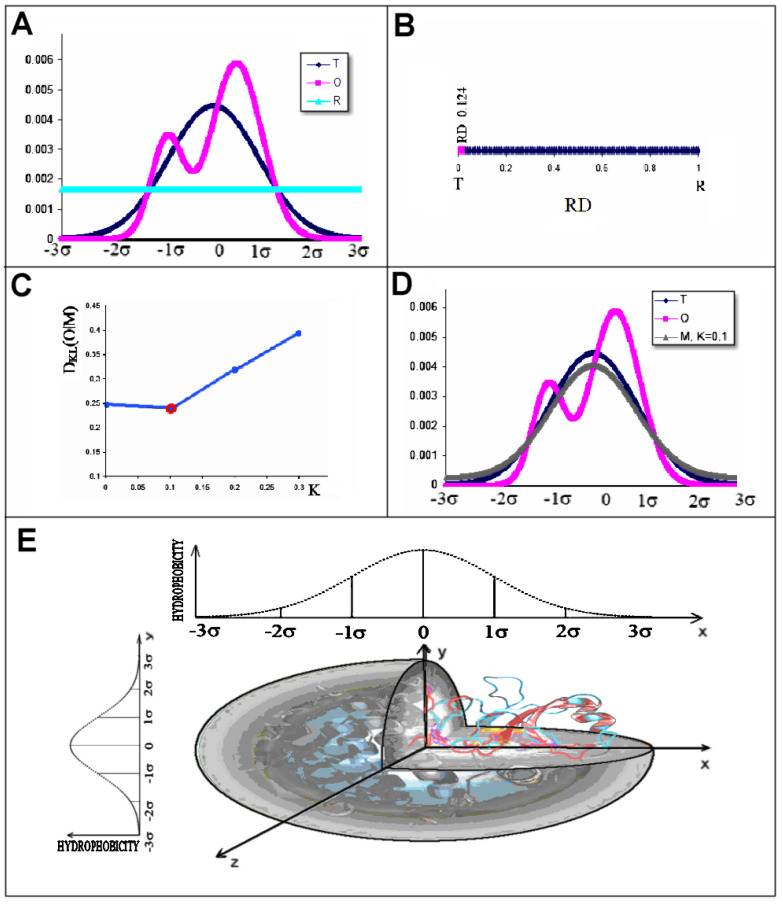
Illustration of the applied model in a presentation simplified to 1D. (**A**)—set of the distributions expressing the hydrophobicity distribution (reduced to 1D presentation for simplicity. This distribution is generated arbitrarily as independent on the 3D analysis.) The vertical axis represents hydrophobicity level: T (blue), O (pink), and R (turquoise). (**B**)—RD value determined for this set on the scale. The position of T value on the scale denotes the perfect T distribution; the position of R on the scale denoted the distribution perfectly accordant with R—it means all residues are carrying equal level of hydrophobicity in protein under consideration. (**C**)—determination of the K parameter value (Equation (6)). The minimum value of D_KL_(O|M) is highlighted. (**D**)—set of the distributions of T (blue), O (pink), and M (grey) for the K value indicated in the legend. The vertical axis represents the hydrophobicity level. (**E**)—protein encapsulated in 3D Gauss function and localised in coordinate system with two projections (according to axis X and Z) assuming the Gaussian distribution of hydrophobicity.

The idealised hydrophobicity distribution representing the presence of a centric nucleus was expressed by a 3D Gaussian function. The amino acids present in a protein were described by the function’s values at the positions of the ‘effective atoms’ (the averaged position of the atoms present in an amino acid) expressing the expected, idealised level that would be present at a given point assuming an arrangement compatible with the hydrophobic nucleus and polar shell present. The function expressing the position on the *i*-th effective atom had the following form:

(1) $$H_{i}^{T}=\frac{1}{H_{sum}^{T}}\exp\left(\frac{-{\left(x_{i}-\bar{x}\right)}^{2}}{2\sigma_{x}^{2}}\right)\exp\left(\frac{-{\left(y_{i}-\bar{y}\right)}^{2}}{2\sigma_{y}^{2}}\right)\exp\left(\frac{-{\left(z_{i}-\bar{z}\right)}^{2}}{2\sigma_{z}^{2}}\right)$$

where *x_i_*, *y_i_*, and *z_i_* are the coordinates of the i-th effective atom. The parameters *σ_X_*, *σ_Y_*, and *σ_Z_* express the size of the protein size-adapted ellipsoid. This distribution is shown in Figure 3A,D as a blue line. The initial orientation of protein molecule requires the positioning of geometric centre in position (0,0,0) in the coordinate system. The visualisation of protein encapsulation in the 3D Gauss function is shown on Figure 3E.

The actual distribution of hydrophobicity was the result of inter-residual hydrophobic interactions expressed by a function proposed by Levitt [59]:

(2) $$H_{i}^{O}=\frac{1}{H_{sum}^{O}}\sum_{j}\left\{\begin{array}{l} \left(H_{i}^{r}+H_{j}^{r}\right)\left(1-\frac{1}{2}\left(7{\left(\frac{r_{ij}}{c}\right)}^{2}-9{\left(\frac{r_{ij}}{c}\right)}^{4}+5{\left(\frac{r_{ij}}{c}\right)}^{6}-{\left(\frac{r_{ij}}{c}\right)}^{8}\right)\right)\;for\;r_{ij}\leq c\\ 0,\;\;for\;r_{ij}>c \end{array}\right.$$

where *r_ij_* is the distance between the i-th and the j-th effective atoms, *c* is the cutoff distance, and *H^r^* denotes the intrinsic hydrophobicity of a given amino acid according to hydrophobicity scale applied. Any hydrophobicity scale can be used [60]. *H^o^* indicates the level of observed hydrophobicity. The “*c*” expresses the cutoff distance *c* = 9Å according to [59]. This distribution is shown in Figure 3A,D as a pink line.

The distributions found using Equation (1) ($H_{i}^{T}$) and Equation (2) ($H_{j}^{O}$) are called *T_i_* and *O_i_*, respectively, to express the theoretical and observed hydrophobicity level on the i-th effective atom. These two distributions were compared using the entropy divergence introduced by Kullback–Leibler [61].

(3) $$D_{KL}(P|Q)=\sum_{i=1}^{N}P_{i}\log_{2}\frac{P_{i}}{Q_{i}}$$

where *P_i_* is the *O_i_* distribution, which means the observed distribution (Equation (2)) in this model and *Q_i_* is the reference distribution, which means the *T_i_* distribution in this model (Equation (1)).

The value found using Equation (3) cannot be interpreted directly (this is the value of entropy according to the authors of this definition [61]). Therefore, a second reference distribution inverse to the T distribution was introduced. It was a uniform distribution with equal levels for each effective atom and referred to as R_i_ = 1/N, where N is the number of amino acids in the polypeptide chain. This distribution is shown in Figure 3. A is shown as a green line. This was a distribution presenting an arrangement devoid of a hydrophobic nucleus (Figure 3A). Thus it is opposite to the T distribution. The relation D_KL_(O|R) > D_KL_(O|T) was interpreted as a structure in which a hydrophobic nucleus is present. To eliminate the use of two values for one object, the parameter RD (Relative Distance—Figure 3B) was introduced. It was defined as follows:

(4) $$RD=\frac{D_{KL}\left(O|T\right)}{D_{KL}\left(O|T\right)+D_{KL}\left(O|R\right)}$$

The reference of the O distribution to distributions with a hydrophobic nucleus present (T) and distributions devoid of hydrophobicity level differentiation (R) did not exhaust all possibilities present in the natural environment of living organisms. A different environment is the membrane environment, where in contrast to the 3D Gaussian function, an exposure of hydrophobicity at the surface (for stabilisation in a hydrophobic membrane) and a low level of hydrophobicity in the central part (membrane proteins are very often channels for the transport of various molecules including ions in particular) is expected. Therefore, to describe the target (theoretical, idealised) hydrophobicity distribution in the membrane environment (assuming the directing folding process toward adaptation to hydrophobic environment), a distribution was proposed as expressed by Equation (5):

(5) *M_i_* = *T_max_* − *T_i_*

where *T_max_* expresses the maximum of T distribution (3D Gauss function) calculated for molecules under consideration (Equation (1)). An analysis of numerous proteins including membrane proteins identified a form of function to describe the hydrophobicity distribution expressed by Equation (6):

(6) *M_i_* = *T_i_* + [*K* × (*T_max_* − *T_i_*)*_n_*]*_n_*

The K parameter value, which expresses the contribution of the non-polar environment opposing the 3D Gaussian function, was determined by an iterative procedure (Figure 3C). The identification of the optimum K value suggested a shape of the function representing the environment to which the folding chain conforms its structure (Figure 3D).

Equation (6) expresses the sum of the T function, the presence of a hydrophobic nucleus, and the function expressed with Equation (5). The second component present in the definition of the modified external field expresses the contribution of disturbance factors to the arrangement expected according to Equation (1). The degree of contribution of a disturbance factor to the arrangement according to the T distribution is expressed by the K parameter value. A protein whose hydrophobicity distribution is expressed by the function in Equation (6) at K = 0.0 is a protein revealing the undisturbed presence of a hydrophobic nucleus. The higher the K parameter value, the greater the proportion of non-aqueous factors causing disturbance according to Equation (5). The magnitude of the K parameter indicates the degree of deviation from the distribution expressed by the 3D Gaussian function.

The search for a suitable K parameter value involves identifying that value for which D*_KL_*(*O*|*M*(*K*)) is minimal—this is illustrated in Figure 3C. The final solution is illustrated in Figure 3D, where the M distribution is determined for the set of T and O profiles. The M distribution is assumed to represent T distribution for modified external conditions. It is assumed that O distribution is reached as the effect as closely as possible approaches the changed (with respect to water environment) external conditions.

The term “profile” is used as equivalent to “distribution” especially when the probability distribution in the form of a chart is discussed.

It was assumed that the folding protein adapted (conformed) to the environment. In aqueous environments, it seeks to form a structure with the hydrophobic nucleus present (centralisation of hydrophobic residues with polar exposure). In a non-aqueous environment, the folding protein conforms to the external field as expressed by a function of M for the optimal K parameter value.

Determining the values of the RD and K parameters for the proteins of interest would provide an indication of the environment in which the protein folds, adapting to these external conditions leading to a corresponding distribution of hydrophobicity in the protein body.

The interpretation of the parameters was as follows:

1. RD indicates the degree of restoration of the micelle-like arrangement in the hydrophobicity distribution. The hydrophobicity distribution in the micelle structure (RD < 0.5) was treated as idealised and consistent with a centric hydrophobic nucleus and a polar surface. The higher the RD value in the range [0–1], the more the hydrophobicity distribution in the protein body approaches an aligned distribution with a uniform layout of comparable hydrophobicity levels. This means deprivation of the hydrophobic nucleus.
2. K indicates the degree to which the environment is different from the polar aqueous environment. The higher the K parameter value, the higher the contribution of non-aqueous factors to the formation of the protein structure from the perspective of hydrophobicity distribution.

The proteins discussed here were described with the values of the RD and K parameters. The interpretation of the results obtained concerns the status of the analysed proteins as stated in Section 1 and Section 2 above.

Using the parameters of the FOD-M model, the status of the structural unit can be determined—whether it is a complex, a chain, or a domain. In this case, the 3D Gaussian function was contoured for this unit individually. This means encapsulating the unit into a 3D Gaussian function form appropriate for it (fitted values of the σ_X_, σ_Y_, and σ_Z_ parameters—Figure 3E).

It was also possible to assess the contribution of a specific fragment of a structural unit (e.g., a single chain in a complex, or a domain as part of a chain). It was possible, too, to specify the status of an arbitrary part of the chain (e.g., the chain fragments forming the beta-sheet). In these cases, a separate 3D Gaussian function was not generated. The fragment of the T, O, and R profiles for the selected fragment was normalised. For the normalised T, O, and R profile fragments, it was possible to determine the values of the RD and K parameters. They denote the contribution of the selected chain fragment to the structure of the distribution that is present in the structural unit. It is possible, for example, to determine the local contribution of a selected beta-sheet to the structure of a hydrophobic nucleus or to determine the local contribution of the same chain fragment to the disturbed organisation of hydrophobicity distribution within a structural unit.

So far, the analysis identified proteins with very low RD values and K = 0.0. These proteins belonged to the down-hill, fast-folding, and ultra-fast-folding groups [62]. It was shown that the clear majority of domains treated as individual structural units revealed low RD parameter values. This was interpreted as directing the folding of domains as individual structural units towards a micelle-like organisation of hydrophobicity within the domains.

Membrane-anchored proteins revealed a highly variable status [63,64]. The range of K values for proteins (membrane domains) was between 0.9 and 4.0. This implied a significant contribution to the formation of the environment for the folding of these proteins by non-aqueous factors that included the membrane environment in particular.

A source of environmental change for the folding process was also the presence of chaperone proteins, whose influence also varied, resulting in the status of proteins folded with their help expressed by increased RD and K values for the description of the structures of these proteins [65,66,67].

## 3. Results

The structures of proteins classified as serpins showed a significant degree of similarity. According to the CATH classification [53], two domains were found in the protein structure, with 2.30 referred to as mainly beta-roll and 3.30 referred to as alpha–beta two-layer sandwich (Figure 1 and Figure 2). A beta-sheet was present in both domains. Beta-sheet A in D1 played a significant role in serpin function. Therefore, this super-secondary structural motif was analysed in all the forms of serpin discussed here.

The structure of the D2 domain to which the RCL segment belonged was intended to determine the status of this chain segment in the protease inhibition process. Detailed description of experimental analysis of serpin activity can be found in [68,69,70,71,72,73,74,75,76,77,78,79,80,81,82].

### 3.1. Status of Complexes and Chains Present in Them

A summary of the parameters describing the status of complexes, the chains treated as components of complexes and the assessment of the status of chains treated as individual structural units (with a 3D Gaussian function generated for each chain individually) in all serpin structural forms discussed here is provided in Table 3, allowing a comparative analysis with an indication of the structural role of individual chains.

The RD values determining the status of the complexes showed a significant mismatch between their hydrophobicity distribution and that expected for the aqueous environment (due to high RD and K values). The status of the individual chains included in the complexes indicated a significant mismatch, especially of the protease chain and, in particular, in the covalent serpin–protease complex. This was mainly due to the reciprocal arrangement of the chains comprising the complex (Figure 1 and Figure 2), which introduced a significant asymmetry in the arrangement (making it remote from a globular arrangement).

Analysis of the T, O, and M profiles (for the corresponding K value) representing the structure of the complexes revealed the location of those sections that did not conform to the idealised reference arrangement represented by the T distribution.

**Figure 4:**
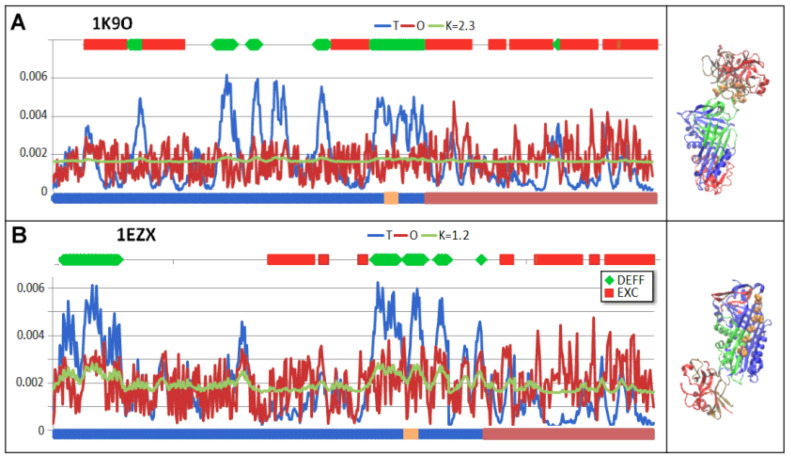
Set of the T, O, and M profiles for the K value given in the legend. (**A**)—Michaelis complex (1K9O). The protease chain (ochre) and serpin (blue) are highlighted on the lower axis. In addition, the locations of RCLs are highlighted in orange. The colour scale on the top axis reveals the locations of local excess—red, and deficiency—green. (**B**)—covalent serpin–protease complex (1EZX), ref. to the colour coding specified for (**A**). The profile sets are accompanied by a 3D structure with colour-differentiated sections revealing the status versus the idealised distribution. Ref. to the colour coding specified for (**A**). The RCL section in the 3D presentation is highlighted with orange space-filling graphics.

A local exposure of hydrophobicity (sections highlighted in red) and a local deficit of hydrophobicity (sections highlighted in green) can be seen (Figure 4).

The locations of areas revealing a hydrophobicity deficit is a natural consequence of the asymmetrical arrangement of the complexes (a disturbed globular structure) caused by the presence of protease.

Areas showing exposure of hydrophobicity are the result of a linear arrangement of interconnected chains. Protease, which shows self-adaptation to the micelle-like arrangement (Table 3) in the complex arrangement caused in a local excess of hydrophobicity in particular (Figure 4).

According to an interpretation based on the FOD-M model, the variation in the presence of areas of hydrophobicity deficit and excess might represent a signal for the arrangements responsible for digesting the complexes in question. The presence of such variation resulted in a differential ordering/disordering of the water molecules, which could be identified by the relevant proteins partial to downstream processes.

### 3.2. Structure of the Serpin Chain

**Figure 5:**
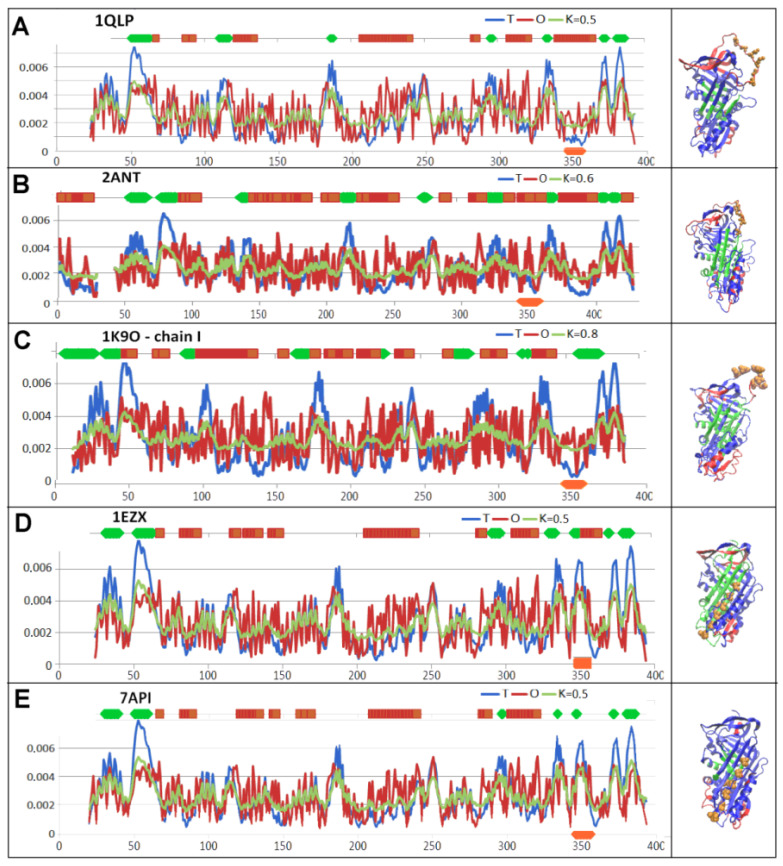
Status of the serpin chain treated as an individual structural unit (with a 3D Gaussian function generated for the serpin molecule only) expressed by a set of T, O, and M profiles for the corresponding K value (provided in the legend for each set). The top horizontal lines indicate the status of the section in question: green—local hydrophobicity deficit, red—local hydrophobicity excess. Similarly, these episodes on 3D presentations were highlighted. The lower horizontal axis shows the location of the RCL—orange. Using the orange space filling form, the position of the RCL in the 3D presentation is indicated. (**A**)—native form (PDB ID—1QLP). (**B**)—latent form (PDB ID—2ANT). (**C**)—Michaelis complex (PDB ID—1K9O). (**D**)—covalent serpin–protease complex (PDB ID—1EZX). (**E**)—cleaved form (PDB ID—7API).

The status of the individual chains treated as individual structural units (with a 3D Gaussian function generated for each chain individually) revealed adaptation to the conditions of the aqueous environment—with the exception of the latent and Michaelis complex forms, where the RD and K values were respectively overestimated (Table 3, Figure 5A–E). This was due to the exposure of the RCL (Figure 5C). In contrast, the status of the protease irrespective of the form of interaction with serpin was comparable and as expected for an aqueous environment—a distribution consistent with that of a centric hydrophobic nucleus and a polar surface (Table 3).

The status of serpin, irrespective of the structural form in which it was present, revealed minimal over-discrimination (RD = 0.5) which was related to the function of the protein. This status was appropriate for the aqueous environment in which serpin is active. An arrangement with a hydrophobicity distribution fully corresponding to a micelle-like arrangement did not show any preparation for a specific interaction. Therefore, the presence of a mismatch—including a local one in particular—with the micelle-like arrangement is a code for writing the specificity of a given protein.

In its native form, the RCL revealed a local hydrophobicity excess, making it ready for interaction. The RCL had a similar status in the Michaelis complex form, where the RCL exposure resulted from the RCL interaction with protease. Elsewhere the latent, covalent serpin–protease complex and cleaved status of the RCL as a component of beta-sheet A followed a T distribution showing a contribution to the hydrophobic nucleus structure.

The hydrophobicity deficit in the central part encompassing beta-sheet A indicates possible structural changes to it. This deficit was present in all the structural forms discussed, yet to varying degrees. The deficit was significantly lower compared to the corresponding deficit found in the complexes.

### 3.3. Status of the Domains

The status of the domains in all compared structural forms of the serpin in question was comparable (Table 4). The D1 domain without incorporated additional sections revealed very high ordering with low K values. This indicated the achievement of domain stability suitable for aqueous environment conditions.

Incorporation of the latent, covalent serpin–protease complex and cleaved sections did not significantly alter the status of D1 domains in the structural and functional forms of serpin discussed.

Similarly, the D2 domain in all forms revealed significant alignment with the micelle-like arrangement, which contributed to the stability of this domain.

The main element of the super-secondary structure in beta-sheet A, as a component of the D1 domain—as expressed by the RD value—revealed levels above the RD = 0.5 discrimination level with the exception of the Michaelis complex form, where the beta-sheet status in the D1 domain was expressed by a value below RD = 0.5 with a very low value of the K parameter. Incorporating the relevant sections into the beta-sheet changed the status of the latter slightly while retaining comparable values of the K parameter.

The beta-sheet B in D2 had an ordering expressed by low K values except for the Michaelis complex form, where the RD value exceeded the RD = 0.5 discrimination level.

**Figure 6:**
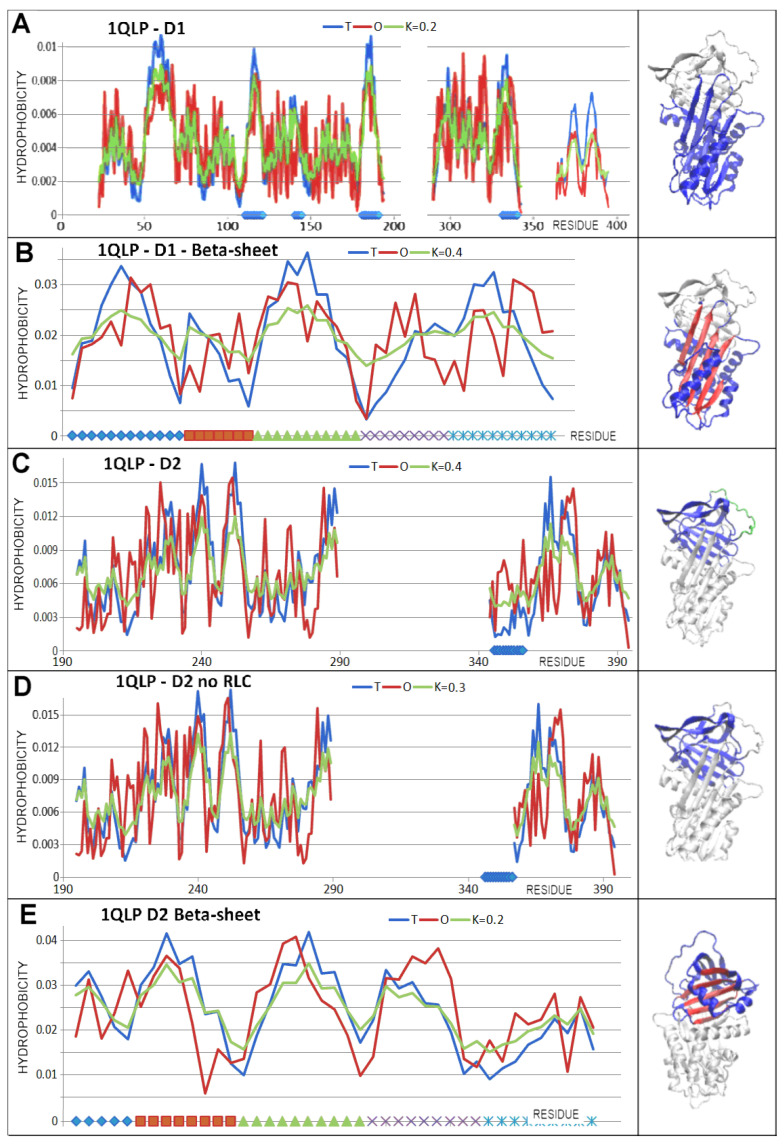
Status of domains as individual structural units and the role of beta-sheet in the domains. (**A**)—T, O, and M profiles describing the status of the D1 domain treated as an individual structural unit—the K value is provided in the legend. (**B**)—beta-sheet A status in D1. (**C**)—T, O, and M profiles describing the status of the D2 domain treated as an individual structural unit—the K value is provided in the legend. The RCLs are highlighted in green. (**D**)—T, O, and M profiles describing the status of the D2 domain treated as an individual structural unit after removal of the RCL—the K value is provided in the legend. (**E**)—beta-sheet B status in D2 (red) specified for the beta-sheet treated as an individual unit.

A detailed analysis of the D1 domain status shown in Figure 6 illustrates the high adaptation to micelle-like distribution in the D1 and D2 domains of the native form. This could be interpreted as the feasibility of independent and spontaneous folding under the influence of the water environment.

Of particular note is the assessment of the RCL status, which plays a role in the assessment of the D2 domain structure (Table 4 and Figure 6).

**Figure 7:**
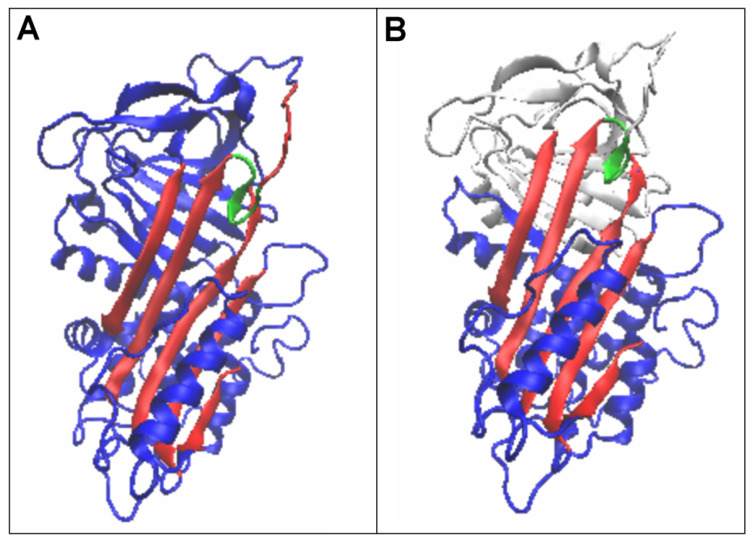
Three-dimensional presentation of the latent form of serpin. A short section of the RCL anchored in beta-sheet A is highlighted (green). (**A**)—complete serpin molecule with beta-sheet A highlighted (red) and the part of the RCL designated in Table 4 as X and anchored in beta-sheet A (green). (**B**)—3D beta-sheet A structure (red) with the attached RCL chain fragment (green—ref. Table 4 designation X) as a component of beta-sheet A. The D2 domain is in white.

**Figure 8:**
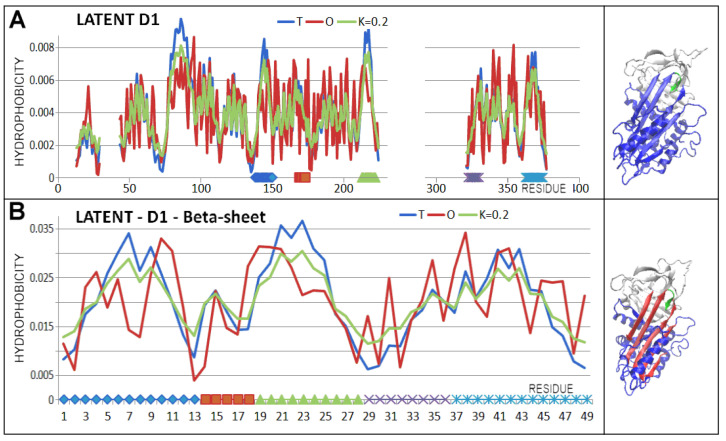
D1 status in the latent form of serpin. (**A**)—T, O, and M profiles (with the K value specified in the legend) together with a 3D presentation. A RCL chain fragment is highlighted (green)—the horizontal bright green line at the bottom of the profiles and the green section of the chain in the 3D representation. The other sections highlighted in colour are beta-sheet A components. (**B**)—beta-sheet A status including presence of the RCL chain fragment (green solid line on the horizontal bottom line) is not in agreement with the table.

The latent structure similarly represents a status very close to a micelle-like arrangement. After incorporation of the RCL section (Figure 7—green fragment; Figure 8), the D1 domain was given a status with reduced RD and K values.

The latent status of the D2 domain revealed the highest degree of deviation (compared to analogous assessments in the other structures) from the micelle-like arrangement (a higher RD value), although the K value remained low (Table 4, Figure 6).

Of special note is the latent form, where a short section of the RCL is spliced into beta-sheet A (Figure 7 and Figure 8). This arrangement is referred to as meta-stable or even unstable intermediate (M*) [54]. The structural status of the complete chain as well as the D1 domain for this structural form was described by slightly higher RD and K values compared to the status of the native form. However, this does not imply a radical change in status versus the native form.

**Figure 9:**
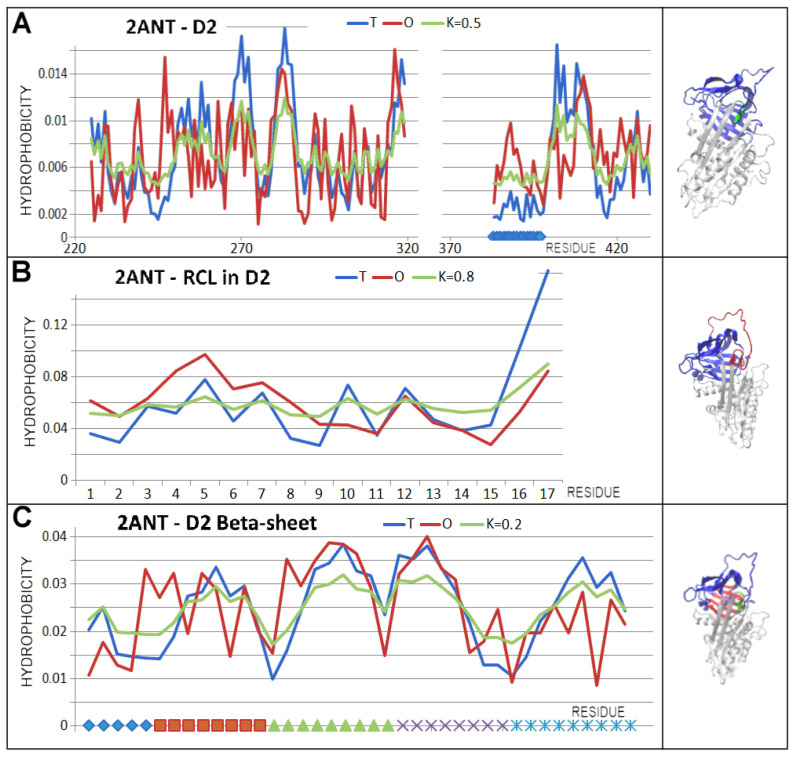
D2 domain status. (**A**)—T, O, and M profiles for the D2 domain. (**B**)—profiles describing the status of the RCL treated as a chain fragment of the D2 domain. See the red fragment in the 3D presentation. (**C**)—profiles describing the beta-sheet B status, together with a 3D presentation where this sheet is highlighted in red. On the horizontal axis, successive beta-sheet sections were differentiated accordingly. Beta-sheet in D2 called Beta-sheet B is distinguished as red in (**C**).

**Figure 10:**
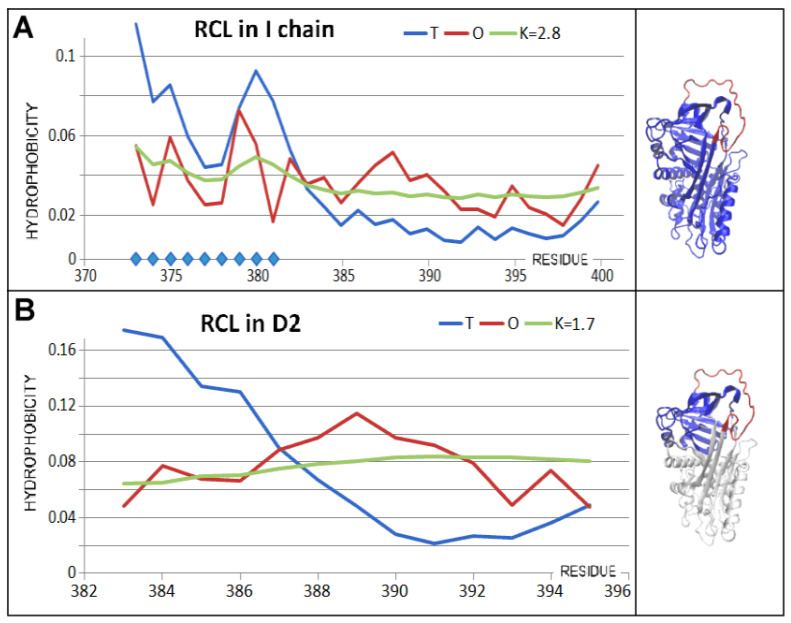
RCL status. (**A**)—T, O, and M profiles for the RCL including the section incorporated into beta-sheet A (red in the 3D presentation) treated as part of the overall chain. (**B**)—T, O, and M profiles for the RCL treated as a component of the D2 domain. See the red highlight in the 3D presentation.

Significantly higher values of the RD and K parameters were revealed in the D2 domain, which was associated with a change in the location of the part of the RCL. The status of domains treated as individual structural units was comparable to the analogous one in the native form. Here, the RCL with a beta-structure section incorporated into beta-sheet A received special treatment. The presence of this section slightly affected the status of the parts of the chain structure in question compared to the status of the native form (Figure 9 and Figure 10).

The M^*^ form, in the light of the analysis based on the FOD-M model, revealed a slightly less structured status in the sense of the FOD-M model. The change in value did not affect the radically different interpretation of the status of both the domain and beta-sheet present in them.

**Figure 11:**
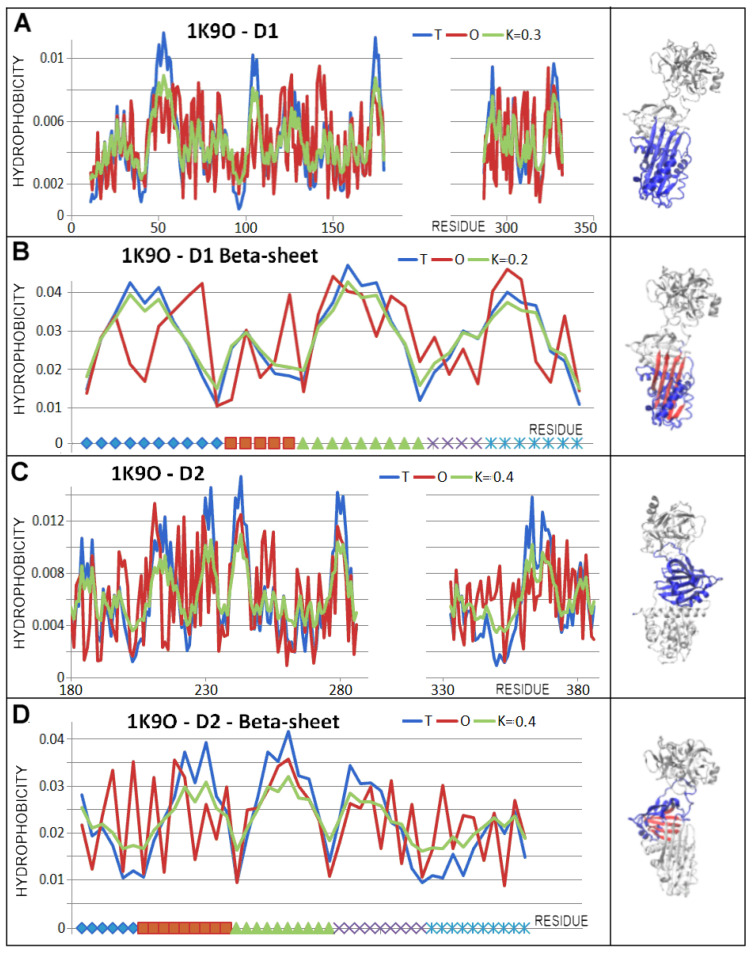
Status of the Michaelis complex. (**A**)—set of T, O, and M profiles showing the status of the D1 domain treated as an individual structural unit—blue in the 3D presentation. (**B**)—status of the beta-sheet A treated as an independent structural unit—red in the 3D presentation. (**C**)—status of the D2 domain treated as an individual structural unit—blue in the 3D presentation. (**D**)—status of beta-sheet B, denoted by a set of T, O, and M profiles, expressed as an independent structural unit. Beta-sheet called B (in D2 domain) distinguished as red in (**D**).

The status of domains in the Michaelis complex form differed from the other structural forms analysed (Table 4 and Figure 11).

The status of domains within the serpin structure was comparable to the native form both for domains treated as components of the chain structure and treated as individual structural units. In contrast, the role of beta-sheet A in the structure of the D1 domain appeared to be ordering the hydrophobicity distribution as understood in the FOD-M model.

**Figure 12:**
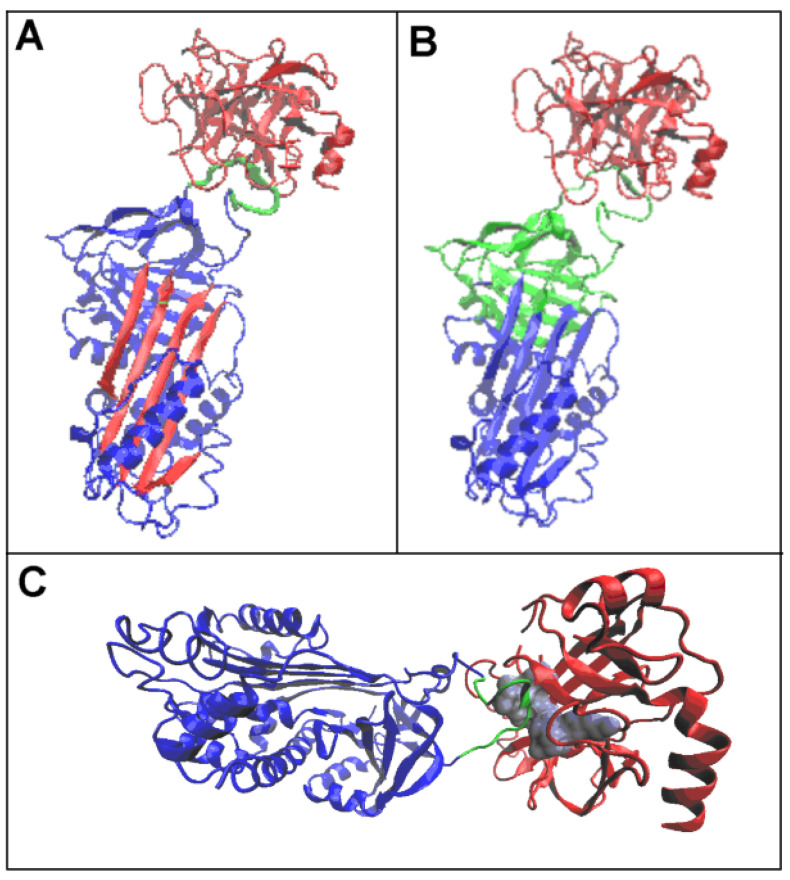
Three-dimensional presentation of the Michaelis complex structure (PDB ID—1K9O). (**A**)—serpin (chain I) in blue with a chain fragment of RCL (346–356) in green and trypsin (chain E) in pink. (**B**)—3D presentation of the Michaelis complex with the D2 domain in green and protease in pink. (**C**)—3D presentation of the Michaelis complex with serpin in blue, protease in red, RCL in green, and catalytic residues of protease as a grey space filling the presentation.

The serpin chain status of the Michaelis complex revealed elevated RD and K values compared to the native form (Figure 11 and Figure 12 and Table 4). The status of the part of the serpin devoid of the RCL chain fragment revealed even higher RD and K values, indicating that the contribution of the RCL is important in ordering the hydrophobicity distribution within the serpin. A different status was identified in a serpin devoid of RCL for 1K9O.

The status of the enzyme, protease, showed an ordering consistent with a micelle-like arrangement. The presence of the RCL chain fragment reduced RD at a constant K value in both structures of interest.

This implies that, in the mechanism of interaction between serpin (RCL) and protease, there is a significant contribution of the complement of hydrophobicity distribution of the enzyme approximating the distribution to a micelle-like distribution.

The RCL section therefore has a doubly stabilising role in the arrangement of the complex. On the one hand, the presence of the RCL section in the serpin structure affected the micelle-like ordering. The presence of the RCL section treated as a component of the protease–RCL complex also improved the ordering as understood in the FOD-M model.

According to reports describing experimental observations, the fast-forming complex leads to the active form, which is called the Michaelis complex in this discussion [53,54,55,56,57]. If the process of formation of the protease activity inhibiting complex is slower, there is cleavage caused by protease on the RCL chain, with the formation of a covalent bond between protease and the position termed P-P’ in the RCL chain. This course of reaction results in the formation of a permanent bond between the two proteins. This process resulted in a significant displacement of the RCL, which becomes spliced as an additional beta section into beta-sheet A [56].

The displacement of protease was a radical change in its position from proximity to the D2 domain to a location at the opposite pole of the entire serpin chain structure, with a location below the D1 domain. This change was associated with the incorporation of the RCL chain fragment into beta-sheet A, resulting in an arrangement of six chain fragments. The incorporated chain fragment adapted to the beta-sheet A arrangement to form an additional chain fragment—a component of the sheet. In contrast, the C-terminal chain fragment of the serpin chain (the RCL residue) was incorporated into beta-sheet B, forming an additional component of the latter. This section was identified in the PDB file as chain B. This form of the complex, albeit described by high RD and K values, turned out to represent an ordering with a lower degree of non-conformity to the micelle-like arrangement.

### 3.4. Status of Beta-Sheets

**Figure 13:**
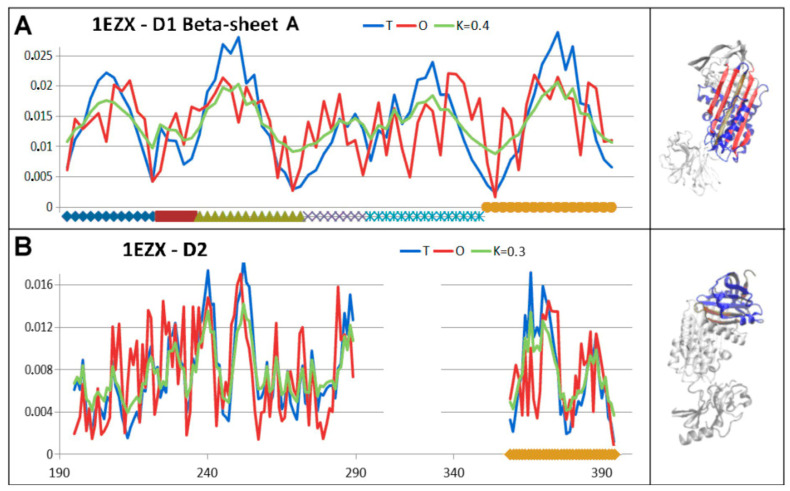
Set of the T, O, and M profiles (for respective K values) describing the status of the domains in covalent serpin–protease complex (PDB ID 1EZX) with a 3D presentation. (**A**)—beta-sheet in the D1 domain; gold is the section incorporated into beta-sheet A on the axis and in the 3D presentation. The red and ochre colours distinguish beta-sheet A in D1 domain. (**B**)—D2 domain status.

The beta-sheet A status with the incorporated additional chain fragment was described by higher RD and K values than for the native structure (Figure 13).

**Figure 14:**
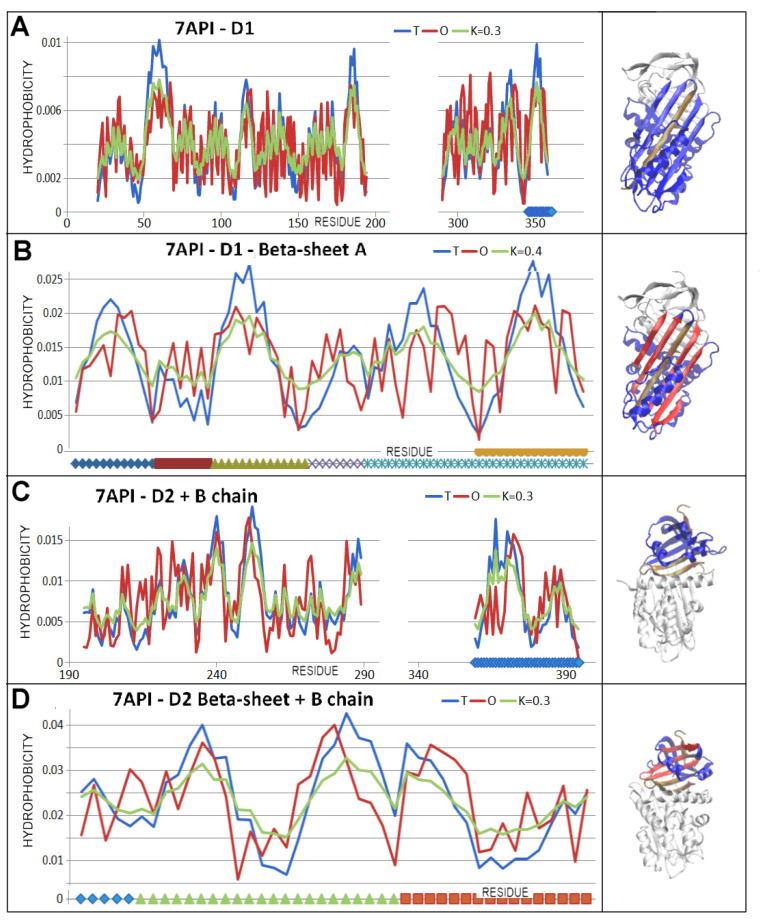
Characterisation of the domains and beta-sheets present in the covalent serpin–protease complex (7API). The RCL C-terminal section is highlighted in gold on the axes of the profile set and in the 3D presentation. (**A**)—T, O, and M profiles for the D1 domain with the RCL chain fragment attached as a beta-sheet A component A—6 beta chain fragments in the sheet. (**B**)—beta-sheet A status with the RCL chain fragment attached (gold). (**C**)—set of the T, O, and M profiles describing the D2 domain status. (**D**)—beta-sheet B status. Beta-sheet called B (in D2 domain) distinguished as red and ochre in (**D**).

The hydrophobicity distribution within the beta-sheets appears to be far from a micelle-like arrangement. This is particularly evident with the beta-sheet A in D1 form present in the covalent serpin–protease complex (Figure 14).

A summary (Table 4) reveals the status of the arrangements in question to be micelle-like. This is probably related to the fact that the environment for serpin activity is the polar environment of water. Hydrophobicity distributions in all forms of the D1 domain are described with RD values below the 0.5 threshold to identify the presence of a hydrophobic nucleus. However, very low K values mean that this structure was produced in conformity with the effect of the aqueous environment. Incorporating additional chain fragments into the D1 domain did not result in significant changes in the assessment of the hydrophobicity distribution ordering, suggesting a mainly aqueous environment is involved. The beta-sheet, which was the central component of the D1 domain structuring, revealed slightly elevated RD values at low K values.

The D2 domain revealed similar characteristics suggesting a structuring resulting from the effect of the aqueous environment (a low K value).

### 3.5. Comparable Analysis of Discussed Structural Forms

**Figure 15:**
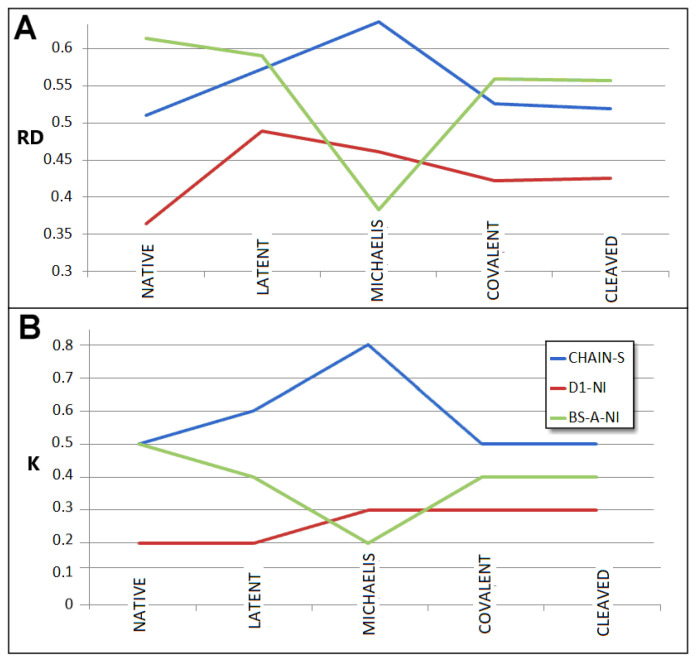
The changes in RD and K values for discussed structural forms (**A**)—RD values; (**B**)—K values for CHAIN. S—serpin, D1-NI—domain 1 without incorporation of additional beta strand. Beta-sheet A, BS-A-NI—Beta-sheet A without incorporation. The charts visualise the data given in Table 3 and Table 4.

The comparable analysis of discussed in this paper is presented in graphic form on Figure 15. It represents the process “native → latent → Michaelis → covalent → cleaved” characterised by the FOD-M model-based parameters. This presentation makes the assessment of the changes possible. The RD parameter of complete chain lovers for latent form with respect to native one reaches its maximum with the Michaelis complex. The two forms, covalent and cleaved, appear comparable in respect to RD parameter. Status of D1 domain (no incorporation) keeps the low RD values for all discussed structural forms (RD < 0.5) with highly comparable status for both forms, covalent and cleaved. Beta-sheet A, relatively high in native form, decreases in latent form with significant lowering of the RD parameter for the Michaelis complex, reaching a level comparable with the whole chain status in covalent and cleaved forms.

The changes in K parameters for compared structural forms appear very similar to RD changes.

High RD and K for the Michaelis complex as shown it Figure 10 is due to significant exposure of RLC loop which makes the chain structure rather unstable from the point of view of hydrophobicity ordering. However, the low RD for beta-sheet A in this form appears the lowest one. It means the stability in this form is kept due to very good order of this super-secondary structural unit.

As it is shown in Table 3 and Table 4, the incorporation of additional beta strand to beta-sheet A causes the small decrease in RD values for covalent and cleaved forms of serpin.

General interpretation of RD values is to express the degree of hydrophobic distribution accordance in respect to idealised 3D Gauss distribution (Equation (1)) The higher RD value, the lower presence of this type of ordering. The RD < 0.5 is interpreted as the presence of hydrophobic core, which generally is treated as III-order structural stability. Increase in RD in comparison of two or more structures indicates the lowering of micelle-like organisation.

The K value expresses the degree of participation of other than water environment. The increase in this value signals the increase in factors other than water present in the environment which influence the folding process. The changes in K values in comparison are of a very low level. The structural changes under consideration are not related to folding–unfolding. The structural changes are of local character in the discussed examples.

The serpin is active in water environment. This is why the K values for all discussed examples are very low and rather stable.

### 3.6. Comparison of T, O and M Distribution with Mobility of Residues in Discussed Structural Forms

To strengthen the interpretability and credibility of the presented model, the comparison of FOD-M outputs with experimentally observed B-factors is included in analysis. The correlation coefficients for T, O, and M distributions with the distribution of B-factors as they are available in appropriate PDB files. The mean values of B-factors for residues were calculated. The correlation coefficients were calculated to express the relation of the hydrophobicity related status of particular residues with respect to their mobility as expressed by B-factor (Table 5).

The results shown in Table 5 present all correlation coefficients as negative values. The high hydrophobicity values are related to the positions in central part. These residues participate in hydrophobic core formation. This is why their mobility is low. The low hydrophobicity level is related to positions near the surface where the mobility may be higher. This is why the relation is inversely proportional.

The same values of correlation coefficients for T and M distributions are obvious due to regular modification of T distribution producing the M distribution.

**Figure 16:**
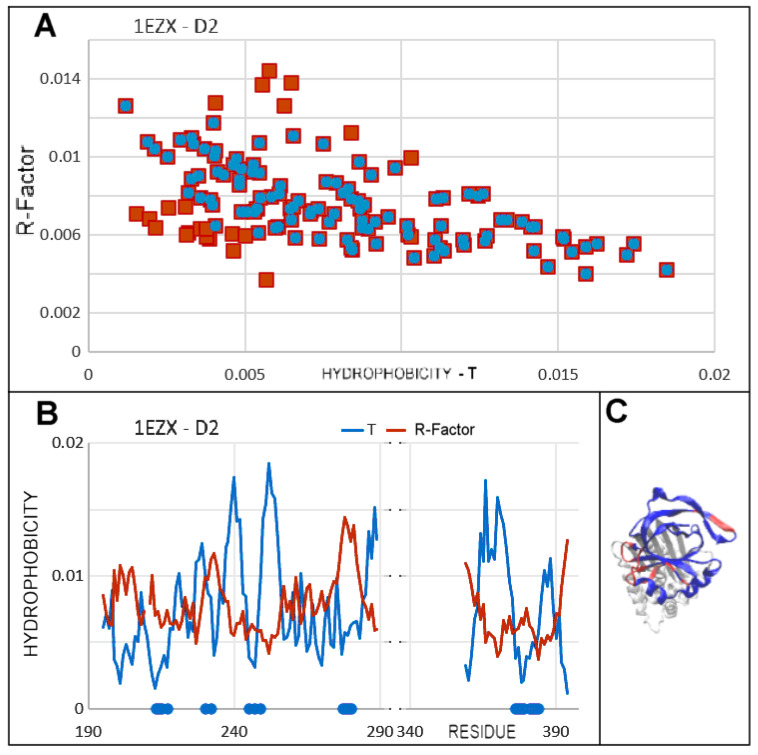
Visualisation of residues treated in correlation assessment as outstanding points. (**A**)—Relation between Ti (x-axis) and B-factor (y-axis) of residues. The outstanding points are distinguished as red. Elimination of outstanding points was repeated step-wise to reach the value of correlation coefficient higher than −0.7. (**B**)—T and O profiles of D2 domain of 1EZX. The positions of residues identified in (**A**) as represented by outstanding points are identified as blue dots on the horizontal axis. Their status with respect to hydrophobicity distribution appears to represent the discordance in form of local excess of hydrophobicity (Oi values higher than Ti). (**C**)—3D presentation of D2 (blue) with residues (red) identified by outstanding points as shown in (**A**). The D1 domain—white.

The example of relation between hydrophobicity level and mobility (expressed by B = factor available in PDB files) shows that the outstanding points represent the residues which do not follow the expected status according to 3D Gauss function. Very frequently such residues are engaged in expression of biological activity. This is shown in Figure 16.

Figure 16A shows the residues elimination (red dots) of which makes the correlation coefficient higher (protein arbitrarily chosen). Their status on hydrophobicity distributions is rather discordant, expressing the local excess of hydrophobicity. The RD value for 1EZX-D2 is equal to 0.444 (Table 4). This value gets lower (RD = 0.401) after removal of the residues identified as outstanding points in Figure 16A. Their status expressed by Ti and Oi can be seen in Figure 16B. These residues are localised in loops exposed on the surface (Figure 16C).

This issue of relation between experimentally observed factors expressing the mobility and form of participation in hydrophobicity distribution presented here is just the preliminary analysis. It requires large-scale research with many proteins as objects of analysis.

## 4. Discussion

The structure of serpins discussed in context of folding and misfolding regards mainly the RCL section [83,84]. The importance of the RCL section has been studied in numerous experiments, mainly in the form of introduced mutations [85,86,87,88,89,90,91]. The result of these studies was to demonstrate the specificity of the RCL in preparation for its variable interaction and stabilisation with the enzyme and with its own beta-sheet. The atypical inhibition mechanism of serpins associated with their own (suicidal) inhibition was an example different from the mechanisms of other inhibition processes mediated by low molecular mass molecules [92,93].

For the process of incorporating the RCL part into beta-sheet A, it can be assumed to be ‘domain swapping’. The difference, however, is that in the case of serpins, the spliced-in chain comes from the same molecule. All the structural versions discussed here suggest the specificity of the serpin molecule in all its structural forms to balance minor structural changes with ordering levels expressed with RD values just above the discrimination level (RD = 0.5). In some structures, the status was determined by RD < 0.5. This means that structures adapted to the conditions of the aqueous environment.

The mechanism for the formation of dimers and polymers made of serpin [93] is the subject of a separate analysis, in which the process is compared to domain swapping [87,94]. In the case of dimers as well as polymers, the incorporation of polypeptide chain segments of monomers was already inter-molecular in nature, in contrast to the mechanism discussed here of incorporation of chain fragments into the own molecule of the protein. The serpin-specific mechanism of protease inhibition, accompanied by significant structural changes, follows the rules of adaptation (conformity) to the conditions of the aqueous environment [95,96,97].

Analysis suggests the speculative hypothesis of making the complexes described by high RD and K (due to hydrophobicity exposure on the surface—acyl enzyme 1EZX) as prepared for possible aggregation with other proteins [98] including degradation factors.

The FOD-M model applied to analysis—in contrast to standard models—takes the hydrophobicity/hydrophilicity aspects as criteria for structural analysis. It may be treated as supplementary analysis to complete the energy-based analyses present in many publications.

## 5. Conclusions

The hydrophobicity distribution in serpin, but also particularly in the domains, retained the ordering with a centric nucleus and a polar shell at the border of discrimination levels (it means the presence of a hydrophobic nucleus at RD < 0.5). The environment in which serpins are active is aqueous. It is therefore important to preserve the rules relevant to this environment.

A non-ordered hydrophobicity distribution (according to the FOD-M model criteria) was present in complexes that featured protease. This apparent mismatch in the form of the presence of cavity (hydrophobicity deficit) and an excess of hydrophobicity on the surface of the complex may be a tell-tale signal for downstream steps in the degradation process of both serpin and protease (the interaction with degrading agents).

The status of the RCL, which locally represented a mismatch of distribution within the native forms along with a very good distribution conformity in relation to protease, provides an argument for the involvement of a hydrophobic effect, which is also present in the serpin (RCL) and protease interaction.

One can speculate that the Michaelis and covalent serpin–protease complex representing the disordered hydrophobicity distribution (especially on the surface) can be the hypothetical code for recognition by degradation factors.

## Figures and Tables

**Table 1:** Summary of the structural forms of serpin associated with the protease inhibition process analysed in this work. AT—antitrypsin.

PDB—ID	Structural Form	Ref.
1QLP	Human native state α1-antitrypsin	[19]
2ANT	Latent AT III (M*)	[55]
1K9O	Michaelis non-covalent complex with anionic trypsine-2	[54]
1EZX	Acyl-enzyme complex with serine protease	[56]
7API	Cleaved	[57]

**Table 2:** Chain fragments participating in beta-sheets constructions. Fragments incorporated distinguished by *.

PDB ID	Domain 1—Beta-Sheet A	Domain 2—Beta-Sheet B
1QLP	112–121	2218–232
	141–145	236–244
	182–190	247–255
	291–298	370–376
	330–340	382–388
2ANT	140–149	256–262
	167–173	268–273
	213–221	278–285
	323–329	408–414
	364–375	419–426
1K9O	101–109	217–222
	129–133	227–233
	172–179	239–246
	287–291	367–372
	326–332	377–383
1EZX	111–121	227–232
	182–193	237–243
	291–298	248–255
	327–340	370–376 *
	344–357 *	382–388 *
7API	110–121	228–232
	141–145	238–244
	182–193	248–255
	291–298	370–375 *
	327–340	381–388 *
	344–357 *	

**Table 3:** Summary of RD and K parameter values for complexes and their constituent chains treated as components of a complex and as individual structural units.

FORM	PDB ID			Chain in Complex				Chain—Individual			
				Serpin		Protease		Serpin		Protease	
		RD	K	RD	K	RD	K	RD	K	RD	K
NATIVE	1QLP							0.510	0.5		
LATENT	2ANT							0.572	0.6		
MICHAELIS	1K9O							0.636	0.8		
ACYL-ENZYME	1EZX	0.787	2.3	0.778	1.6	0.711	1.3	0.526	0.5	0.447	0.4
CLEAVED	7API	0.726	1.2	0.590	0.6	0.779	1.7	0.519	0.5	0.497	0.4

**Table 4:** Summary of the RD and K parameter values for the domain structural forms and beta-sheets of interest. In the forms where the chain fragment was incorporated, RD and K values were given for versions with and without the incorporated chain fragment. COV.S-P.C. denotes covalent serpin–protease complex.

Form	PDB—ID	Domain 1				Domain 2		Beta-Sheet A in D1				Beta-Sheet B in D2	
		No Incorp.		Incorporated				No Incorp.		Incorporated			
		RD	K	RD	K	RD	K	RD	K	RD	K	RD	K
NATIVE	1QLP	0.364	0.2			0.488	0.4	0.613	0.5			0.429	0.2
LATENT	2ANT	0.489	0.2	0.396	0.2	0.578	0.4	0.590	0.4	0.584	0.4	0.483	0.2
MICHAELIS	1K9O	0.461	0.3			0.514	0.2	0.483	0.2			0.598	0.4
COV.S-P.C	1EZX	0.422	0.3	0.426	0.3	0.444	0.3	0.559	0.4	0.548	0.4	0.447	0.2
CLEAVED	7API	0.425	0.3	0.416	0.3	0.446	0.3	0.557	0.4	0.539	0.4	0.481	0.3

**Table 5:** The correlation coefficients characterising the relation between FOD-M model-based parameters (T, O, and M distribution) and mobility of residues (expressed by B-factor) in domains of discussed proteins.

FORM	PDB ID			Distribution	
		Domain	T	O	M
NATIVE	1QLP	D1	−0.494	−0.549	−0.494
		D2	−0.664	−0.491	−0.664
LATENT	2ANT	D1	−0.503	−0.492	−0.503
		D2	−0.469	−0.375	−0.469
MICHAELIS	1K9O	D1	−0.375	−0.204	−0.375
		D2	−0.403	−0.150	−0.403
COV.S-P.C	1EZX	D1	−0.585	−0.578	−0.585
		D2	−0.483	−0.527	−0.483
CLEAVED	7API	D1	−0.509	−0.523	−0.509
		D2	−0.495	−0.576	−0.495

## Data Availability

The potential user has two possible ways to access the program: The program allowing the calculation of RD as well as T and O distribution is accessible upon request on the CodeOcean platform: https://codeocean.com/capsule/3084411/tree (accessed on 20 May 2005). Please contact the corresponding author to get access to your private program example. The application—implemented in collaboration with the Sano Centre for Computational Medicine (https://sano.science, accessed on 20 May 2025) and running on resources contributed by ACC Cyfronet AGH (https://www.cyfronet.pl, accessed on 20 May 2005) in the framework of the PL-Grid Infrastructure (https://plgrid.pl, accessed on 20 May 2025)—provides a web wrapper for the abovementioned computational component and is freely available at https://hphob.sano.science (accessed on 20 May 2025).

## Abbreviations

The following abbreviations are used in this manuscript:

FOD-M	Fuzzy oil drop model—modified
RD	Relative Distance
